# Psychosomatic interplay in postmenopausal osteoporosis: from neuroendocrine mechanisms to structured psychological therapies and long-term management

**DOI:** 10.3389/fpsyt.2026.1906383

**Published:** 2026-08-20

**Authors:** Haixiang Zhang, Jihan Wang, Yangyang Wang, Cuixiang Xu, Zhankui Jin

**Affiliations:** 1 Shaanxi Provincial Key Laboratory of Infection and Immune Diseases, Shaanxi Provincial People’s Hospital, Xi’an, China; 2 Shaanxi Engineering Research Center of Cell Immunology, Shaanxi Provincial People’s Hospital, Xi’an, China; 3 Yan’an Medical College, Yan’an University, Yan’an, Shaanxi, China; 4 School of Physics and Electronic Information, Yan’an University, Yan’an, Shaanxi, China; 5 Department of Orthopedics, Shaanxi Provincial People’s Hospital, Xi’an, China; 6 Shaanxi Provincial Key Laboratory of Basic and Clinical Transformation of Bone and Joint Diseases, Xi’an, China

**Keywords:** bone-mood axis, postmenopausal osteoporosis, psychological therapies, psychoneuroimmunology, psychosomatic interplay, stepped-care model

## Abstract

Postmenopausal osteoporosis (PMOP) is a prevalent chronic condition that severely compromises both the physical and psychological well-being of aging women. Beyond its skeletal implications, PMOP exhibits a profound psychosomatic interplay, intimately linked with elevated rates of depression and anxiety that frequently impair treatment adherence and functional recovery. This psychological vulnerability arises from a complex web of hormonal shifts, fear of fracture, and significant global sociocultural determinants that restrict care access. This comprehensive narrative review synthesizes current evidence through a biopsychosocial framework, critically examining the underlying psychoneuroimmunology (PNI) mechanisms that govern the bone-mood axis. Furthermore, we evaluate evidence-based psychological therapies (including cognitive behavioral therapy and mindfulness-based interventions) and propose an innovative, hierarchical stepped-care model for long-term patient management. Finally, we identify critical gaps in existing pharmacological and behavioral strategies, offering a multidisciplinary roadmap to advance mental health equity and integrated psychosomatic care for aging female populations.

## 1 Introduction

Osteoporosis (OP) is a prevalent chronic skeletal disorder characterized by decreased bone mineral density (BMD) and microarchitectural deterioration of bone tissue, leading to increased bone fragility and fracture risk (1, 2). Among various populations, postmenopausal women are disproportionately affected due to the abrupt decline in estrogen levels following menopause, which accelerates bone resorption and compromises skeletal strength (2, 3). According to the International Osteoporosis Foundation (IFO), one in three women over the age of 50 will experience osteoporotic fractures in their lifetime, underscoring the global burden of this condition (4, 5). Despite evidence showing that OP affects approximately 18.3% of the global population—rising significantly to 30% in postmenopausal women (6, 7)—its extensive psychological impact on this vulnerable group remains disproportionately overlooked in current literature. While the physical consequences of OP, such as fractures, pain, and mobility limitations, are well-recognized, the psychological implications of the disease have only recently gained attention in both clinical and research contexts (8, 9).

Psychological health plays a crucial role in shaping the experiences and outcomes of individuals with OP, particularly among postmenopausal women (10). The chronic and progressive nature of OP, compounded by fear of falls, perceived physical vulnerability, and functional impairment, can significantly impact mental well-being (11). Numerous studies have reported elevated rates of depression, anxiety, and reduced self-esteem among women living with postmenopausal OP (PMOP) (8, 12). These psychological burdens may not only diminish health-related quality of life (HRQoL) but also adversely affect disease management, including medication adherence, physical activity participation, and engagement with rehabilitation programs (13). Emerging evidence suggests that psychological disturbances in PMOP may stem from complex interactions among hormonal changes, cognitive appraisals of illness, lifestyle modifications, social isolation, and comorbid chronic conditions (14). For instance, estrogen withdrawal has been implicated in dysregulation of neurotransmitters such as serotonin (5-hydroxytryptamine, 5-HT) and dopamine, which are closely linked to mood disorders (15, 16). Additionally, the invisibility of OP symptoms in its early stages may contribute to under-recognition and under-treatment of psychological distress, thereby delaying necessary interventions. Despite growing recognition of the mind-body interplay in chronic disease management, psychological health remains an underexplored and undertreated aspect of OP care. Traditional OP management tends to focus on pharmacologic interventions and fracture prevention, often overlooking the emotional and behavioral needs of patients (17). This gap calls for a more holistic and integrated approach that incorporates psychological assessment and support into routine OP treatment, especially in aging females.

**Figure 1:**
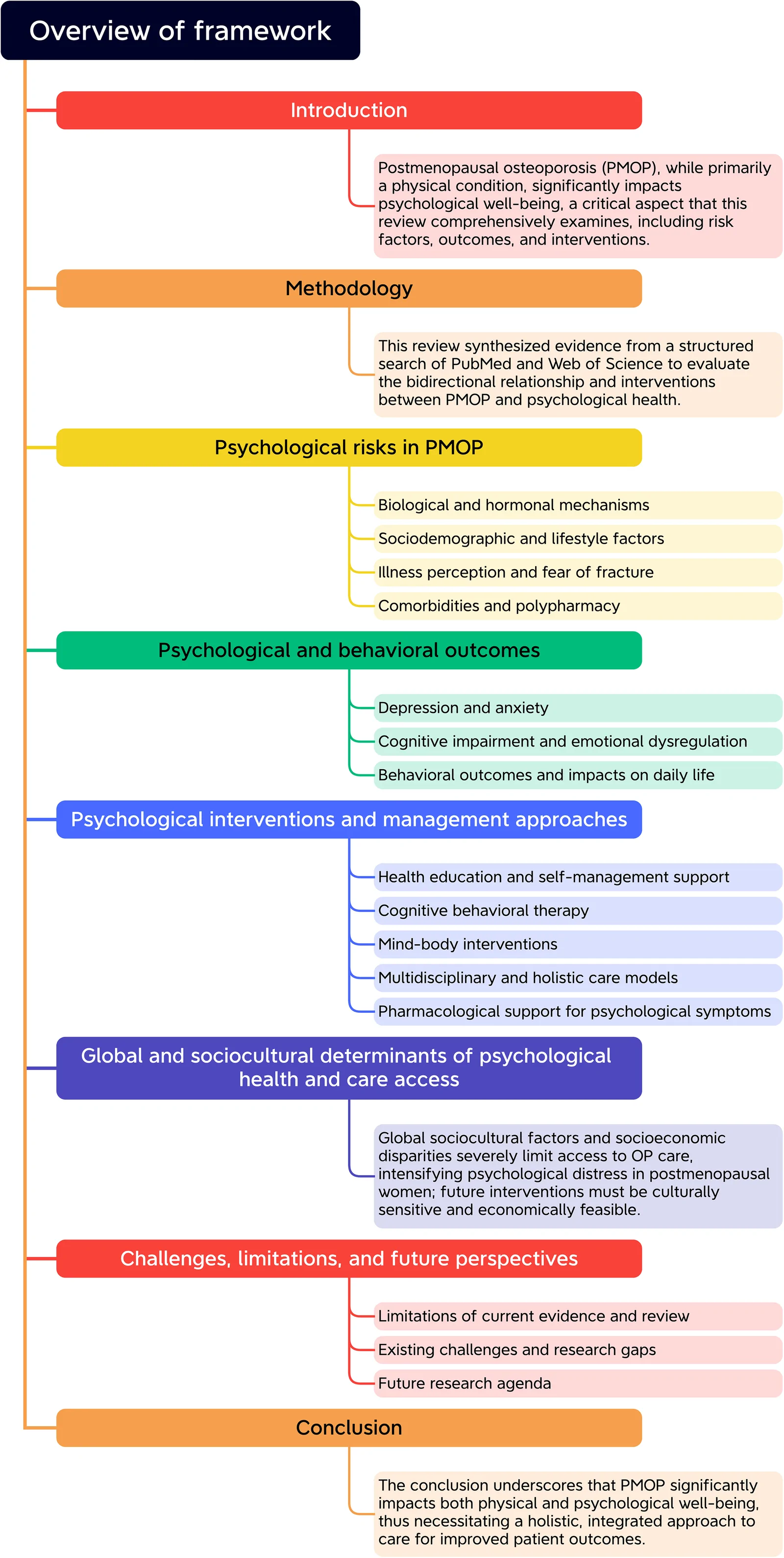
The framework of the review.

**Figure 1** presents the comprehensive framework guiding this review, which synthesizes current evidence on the psychological health of women with PMOP. We explore the multifactorial risk factors contributing to psychological disorders in this population, examine the impact of these conditions on clinical outcomes and quality of life, and evaluate the efficacy of psychological and behavioral interventions. By highlighting the interconnectedness of psychological and physical health in OP, we advocate for an interdisciplinary model of care that addresses both skeletal and mental well-being in postmenopausal women. Despite growing recognition of the mind-body interplay, two critical research gaps persist (1): the lack of standardized psychological screening protocols within routine orthopaedic care, and (2) a limited synthesis of quantitative evidence regarding the bone-mood axis. This review aims to bridge these gaps by providing a biopsychosocial synthesis and proposing a stepped-care model.

## 2 Methodology

To ensure transparency and methodological rigor, this narrative review was conducted through a structured search of PubMed and Web of Science up to June 2025. The search strategy employed explicit search strings, including: (“Postmenopausal osteoporosis” OR “PMOP”) AND (“Depression” OR “Anxiety” OR “Quality of Life” OR “Psychoneuroimmunology”). Inclusion criteria targeted peer-reviewed English articles focusing on PMOP, psychological comorbidities, or therapeutic interventions. Exclusion criteria were non-English publications, conference abstracts without full text, and studies lacking relevance to the bone-mood axis. Out of 1, 420 retrieved records, 126 studies met all eligibility criteria and were synthesized in this review.

## 3 Psychological risks in PMOP

**Figure 2:**
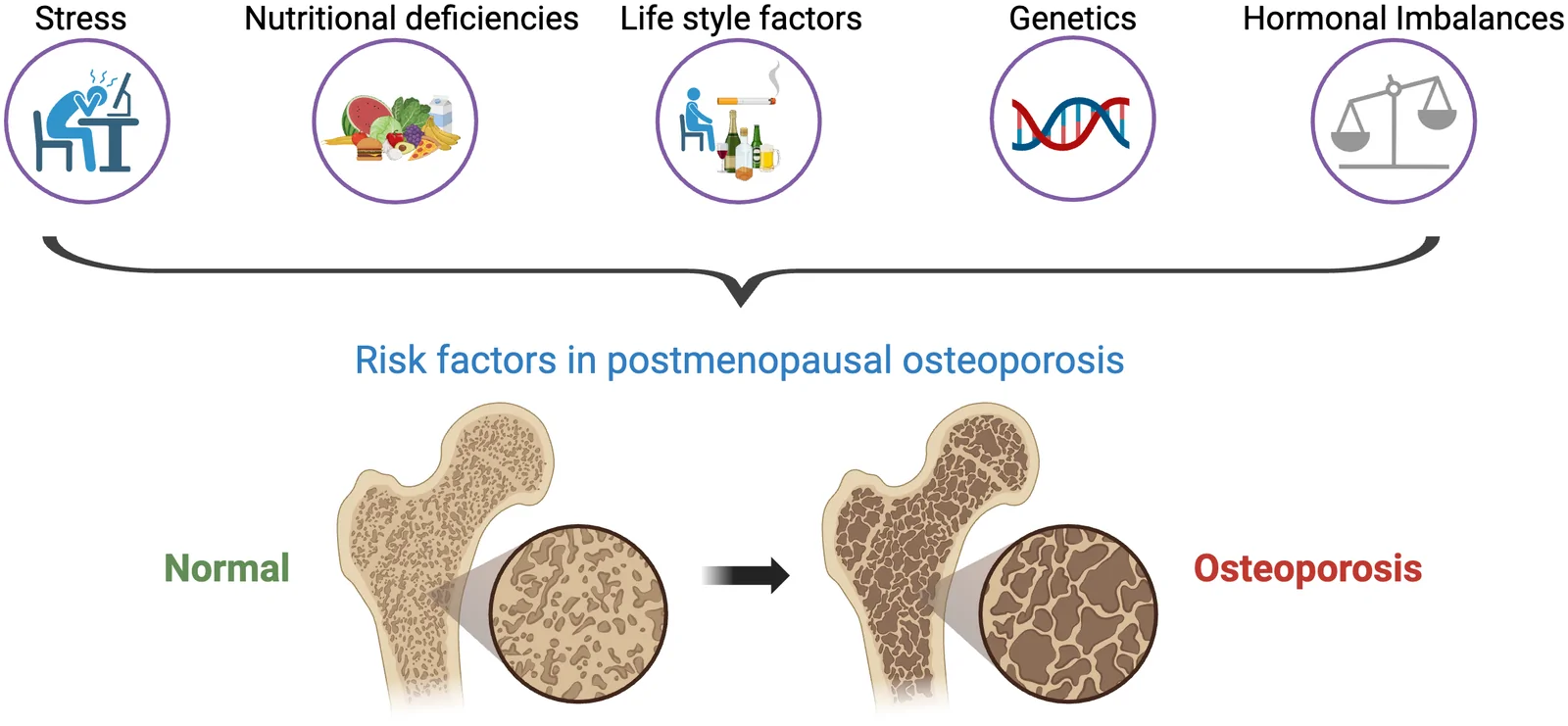
Multifactorial risk factors and integrated mechanisms contributing to psychological distress in postmenopausal osteoporosis (PMOP). The development of PMOP and its associated psychiatric comorbidities arises from a complex interplay of biological, hormonal, and environmental variables. Central to this process is the decline in estrogen levels, which disrupts bone homeostasis and impairs neurotransmitter systems (e.g., serotonin and dopamine) in brain regions responsible for mood regulation. This hormonal shift is further compounded by chronic stress, which activates the hypothalamic-pituitary-adrenal (HPA) axis and increases systemic pro-inflammatory cytokines, creating a feed-forward loop between skeletal degradation and psychological vulnerability. Additionally, lifestyle factors such as physical inactivity, nutritional deficiencies (e.g., Vitamin D and Calcium), and sociocultural determinants like low health literacy further diminish both bone mineral density and psychological resilience in aging women.

Psychological disorders, particularly depression and anxiety, are commonly observed among women with PMOP (18, 19). The development of these conditions is multifactorial, involving the interaction of biological, hormonal, psychological, and social variables (20). Identifying and understanding these risk factors is critical for early detection and the development of targeted interventions. As illustrated in **Figure 2**, key contributors to PMOP and its associated challenges include stress, nutritional deficiencies, lifestyle factors, genetics, and hormonal imbalances. This section explores the major contributors to psychological distress in this population, with a particular emphasis on biological and hormonal mechanisms.

### 3.1 Biological and hormonal mechanisms

The abrupt hormonal shifts that occur during menopause, especially the decline in estrogen levels, are central to both the development of OP and the emergence of psychological disorders (21, 22). Estrogen is not only essential for maintaining bone homeostasis but also plays a pivotal role in modulating brain function and emotional regulation (23). Estrogen receptors (ERα and ERβ) are widely distributed throughout the central nervous system, particularly in brain regions associated with mood regulation, such as the hippocampus, amygdala, and prefrontal cortex (24, 25). The reduction in circulating estrogen levels following menopause is associated with altered serotonergic, dopaminergic, and noradrenergic neurotransmission, which are all implicated in the pathophysiology of mood disorders (26). Serotonin, in particular, is crucial for emotional stability, and its dysregulation has been linked to depressive symptoms in postmenopausal women (27). Building upon these neurobiological links, a large-scale meta-analysis of 21 studies (encompassing 19, 243 individuals) provided robust clinical evidence of the bone-mood axis, demonstrating significant reductions in BMD among patients with depressive disorders. The most pronounced negative effect sizes were identified in the femur (d = −0.34) and lumbar spine (d = −0.15), particularly among female cohorts (28). Estrogen also influences the HPA axis, which regulates the body’s stress response (29). Hypoestrogenism can result in hyperactivity of the HPA axis and elevated cortisol levels—both of which have been independently associated with anxiety, sleep disturbances, and cognitive dysfunction (30). In addition to neuroendocrine changes, inflammation is increasingly recognized as a shared biological pathway underlying both OP and depression (31). Postmenopausal women often exhibit elevated levels of pro-inflammatory cytokines such as interleukin-6 (IL-6), tumor necrosis factor-alpha (TNF-α), and C-reactive protein (CRP) (32). These inflammatory markers not only promote osteoclast activity and bone loss but also interfere with neurotransmitter synthesis and neuronal plasticity, contributing to depressive symptomatology. This systemic low-grade inflammation may act synergistically with estrogen deficiency to exacerbate both skeletal fragility and psychological vulnerability. Moreover, emerging evidence suggests a possible role of vitamin D deficiency—a common comorbidity in PMOP—in the development of mood disorders (33, 34). Vitamin D receptors are present in brain tissue, and low serum levels of 25-hydroxyvitamin D have been linked to increased risk of depression and impaired cognitive performance (35). The overlap between vitamin D deficiency, reduced mobility, and decreased sunlight exposure further compounds the risk of emotional dysfunction in osteoporotic women. In summary, the biological and hormonal landscape of postmenopausal women creates a neurophysiological environment that predisposes them to both OP and psychological disorders.

Beyond systemic hormone shifts, key molecular pathways bridge skeletal dynamics and psychiatric pathology. Estrogen loss and persistent stress upregulate nuclear factor kappa B (NF-κB) and RANK/RANKL signaling while suppressing osteoprotegerin (OPG) and Wnt/β-catenin pathways, accelerating bone resorption. Concurrently, elevated oxidative stress, impaired Nrf2 signaling, mitochondrial dysfunction, and microglial activation in brain tissues suppress brain-derived neurotrophic factor (BDNF)/TrkB signaling, compounding both neurodegeneration and osteoblast apoptosis. These convergent intracellular pathways form the core molecular foundation of the bone-mood interplay.

### 3.2 Sociodemographic and lifestyle factors

Sociodemographic and lifestyle factors significantly impact the psychological well-being of women with PMOP. Low educational attainment often correlates with reduced health literacy, leading to feelings of helplessness and anxiety due to a lack of understanding about disease management, medication, and the importance of exercise and nutrition (36, 37). Socioeconomic status (SES), linked to income, affects access to crucial healthcare services like OP screening, dual-energy X-ray absorptiometry (DXA) testing, and psychological support. Financial insecurity can limit affordability of medications and exercise resources, fostering vulnerability and diminishing self-efficacy. Marital status and social support are also crucial; single, divorced, or widowed women are more prone to loneliness and isolation, increasing depression and anxiety, and potentially leading to reduced physical activity and nutritional neglect. Conversely, strong social support offers emotional reassurance and practical assistance. Physical inactivity is a major concern, as it contributes to both bone loss and mood disturbances, creating a vicious cycle where poor mental health exacerbates inactivity (38, 39). Dietary habits also play a role, with inadequate intake of essential nutrients like calcium and vitamin D affecting both bone strength and mood regulation. Furthermore, excessive alcohol consumption and smoking negatively impact bone metabolism and are linked to higher rates of mood disorders. Recognizing these modifiable sociodemographic and lifestyle factors is crucial for providing holistic OP care, as they profoundly influence psychological health.

### 3.3 Illness perception and fear of fracture

Illness perception—the way individuals conceptualize and emotionally respond to their disease—significantly shapes psychological outcomes in women with PMOP (40). Many women perceive OP as a silent yet progressive threat that may unpredictably lead to disability, deformity, or loss of independence (41). These cognitive appraisals can induce chronic worry, hypervigilance, and emotional exhaustion, especially in those who have already experienced fractures. Fear of fracture, in particular, is a pervasive and well-documented psychological concern (8). Even in the absence of prior fractures, the perceived risk of falling or breaking a bone can lead to increased anxiety, social withdrawal, and avoidance of physical activity. This behavioral restriction not only accelerates muscle deconditioning and bone loss but also reinforces a sense of vulnerability and loss of control (19). Such avoidance behaviors are strongly associated with reduced HRQoL and increased depressive symptoms. Moreover, illness-related fear is often compounded by inadequate information or misunderstanding about the nature of OP and its management. Women who believe the condition is untreatable or inevitably disabling may feel hopeless or disengaged from care, reducing treatment adherence and motivation to adopt healthy behaviors.

### 3.4 Comorbidities and polypharmacy

Comorbid medical conditions are highly prevalent in PMOP women, significantly contributing to their psychological burden (7). Chronic diseases like cardiovascular disease (CVD), diabetes, and osteoarthritis frequently coexist, compounding physical limitations such as pain and fatigue, which negatively impact mental health. Beyond physical symptoms, managing multiple chronic illnesses can lead to profound psychological tolls, including frustration, exhaustion, and helplessness. Navigating numerous healthcare providers and complex treatment regimens often overwhelms these women, especially those with limited support, reducing their self-efficacy and increasing vulnerability to anxiety and depression (42). Polypharmacy, the use of five or more medications concurrently, is also common, adding to distress. Patients take drugs not only for OP but also for comorbid conditions, increasing risks of adverse reactions, interactions, and non-adherence. Furthermore, some medications (e.g., corticosteroids, beta-blockers) can have neuropsychiatric side effects like mood disturbances or cognitive impairment (43, 44). The cognitive burden of managing intricate medication schedules can lead to errors, health management anxiety, and a sense of lost autonomy, cumulatively impacting well-being and treatment engagement. Comorbidities and polypharmacy significantly amplify the risk of psychological disorders in this population, necessitating a multidisciplinary, patient-centered approach that includes mental health screening, medication review, and coordinated care to minimize both physical and psychological complications.

## 4 Psychological and behavioral outcomes

**Figure 3:**
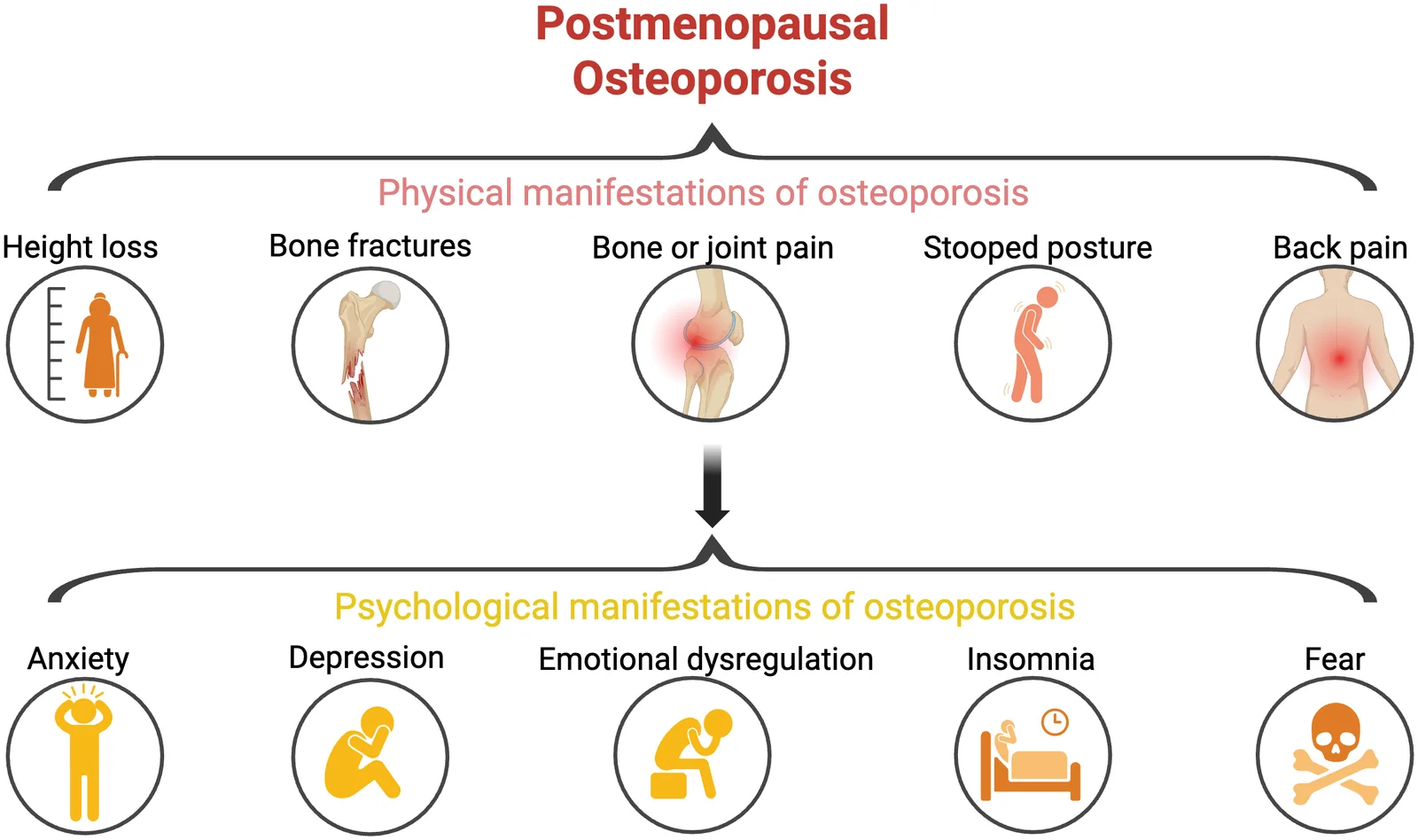
Bidirectional relationship between the physical manifestations and psychological symptoms of postmenopausal osteoporosis (PMOP). PMOP imposes a dual burden where skeletal impairment and mental health outcomes are deeply intertwined. Physical manifestations, including vertebral fractures, loss of height, stooped posture, and chronic pain, directly contribute to functional limitations and a loss of independence. These somatic changes often trigger or exacerbate psychological symptoms, most notably “fear of falling” and fracture-related anxiety, which lead to hypervigilance and social withdrawal. The resulting depression and sleep disturbances further impair the patient’s HRQoL, necessitating a transition from traditional pharmacological focus toward a comprehensive biopsychosocial care model that addresses both the visible and invisible aspects of the disease.

The psychological impact of PMOP women is multifaceted, encompassing emotional disturbances, cognitive dysfunction, and behavioral maladaptation (45). Emerging evidence from systematic reviews suggests that these outcomes are not merely secondary reactions to a physical illness but are integral to the disease experience itself. As comprehensively displayed in **Figure 3**, PMOP manifests through a range of physical symptoms and profound psychological impacts. These arise from the interplay of biological vulnerability, personal coping mechanisms, and sociocultural factors, and they significantly influence both individual well-being and clinical outcomes. Understanding these multifaceted outcomes is essential for transitioning from traditional pharmacological management to more robust, integrated biopsychosocial care models.

### 4.1 Depression and anxiety

Depressive symptoms are highly prevalent in PMOP women. Cross-sectional descriptive studies report the prevalence of these symptoms to be significantly higher among PMOP women compared to age-matched controls (46, 47), with one study also finding depression to be associated with substantially lower BMD (46). A recent study by Cui and Su reported that 73.3% of affected women exhibited depressive symptoms, identifying low BMD, chronic comorbidities, and reduced serotonin levels as key risk factors (48). In addition, a large-scale prospective cohort study enrolling over 93, 000 women demonstrated that both depressive symptoms and antidepressant therapy independently increased the risk of certain osteoporotic fractures (49). Common manifestations include persistent low mood, feelings of worthlessness, reduced energy, difficulty concentrating, anhedonia, and disturbances in sleep and appetite. In more severe cases, these symptoms may progress to clinical depression requiring psychiatric intervention (14). Several mechanisms account for this increased susceptibility. Beyond the decline in estrogen levels affecting serotonergic and dopaminergic neurotransmission, reduced 5-HT levels serve as a critical biochemical link, as this neurotransmitter acts as a dual-regulator of both bone remodeling and mood stability. Moreover, OP itself is often perceived as a symbol of aging, frailty, and decline, evoking existential fears and negative self-perception (50). For women who suffer osteoporotic fractures—particularly of the spine or hip—the physical disability and prolonged recovery can be traumatic, further compounding depressive symptoms. Anxiety disorders, particularly generalized anxiety and health-related anxiety, are also frequently observed. The fear of falling or sustaining a fracture can be pervasive and paralyzing. This fear is not unfounded—osteoporotic fractures are a leading cause of morbidity and mortality among older women. Furthermore, prospective cohort studies have demonstrated that sustained depression and anxiety are independently associated with an increased hazard ratio for subsequent osteoporotic fractures (51, 52). However, when this fear becomes excessive, it can result in hypervigilance, avoidance of daily activities, and social withdrawal. Such anxiety not only impairs quality of life but also reduces engagement in preventive behaviors like physical activity, thus worsening physical health outcomes (53). In some cases, women may develop somatic symptom disorders, characterized by persistent and excessive worry about bodily sensations and health status. These psychological states often go unrecognized and untreated in routine OP care (54, 55). To address this, integrating simple, scalable strategies such as routine screening and nurse-delivered emotional support is essential to bridge the gap between physical and psychological health.

Critically, this psychosomatic interaction is bidirectional. While PMOP-induced functional decline elevates psychological distress, primary depression independently impairs bone health. Chronic depressive states trigger persistent HPA axis hyperactivation, hypercortisolemia, and elevated pro-inflammatory cytokines that disrupt bone remodeling. Furthermore, depression-associated physical inactivity, nutritional neglect, and secondary effects of psychotropic medications further accelerate bone mineral density loss, establishing a self-reinforcing vicious cycle.

### 4.2 Cognitive impairment and emotional dysregulation

Evidence from systematic reviews and meta-analyses has established a bidirectional association between OP and cognitive impairment. Patients with OP have an increased risk of cognitive impairment [Odds Ratio (OR) = 2.01; 95% CI 1.63–2.48] (56), and conversely, patients with cognitive impairment have a significantly increased risk of developing OP [Relative Risk (RR) = 1.56; 95% CI 1.30–1.87] (57). Notably, this risk is particularly pronounced in patients with Alzheimer’s disease, showing a 1.7-fold increased risk of OP (RR = 1.70; 95% CI 1.23–2.37). This impairment often affects domains such as attention, verbal memory, and executive functioning (56). The complex mechanism underlying this reciprocal relationship can be modeled under the framework of psychoneuroimmunology (PNI), which provides a unified link between the hormonal, neurotransmitter, and immune systems (58–60). Psychological distress and chronic stress lead to the dysregulation of the HPA axis, resulting in elevated glucocorticoids and systemic low-grade inflammation (61, 62). This cascade links mood, cognition, and bone health, as pro-inflammatory cytokines simultaneously disrupt neural function and promote bone resorption (63, 64). While the exact mechanisms remain under investigation, proposed pathways include estrogen deficiency, chronic inflammation (65), and shared comorbidities like CVD and diabetes, whose risk is often proportional to OP severity (66, 67)—all of which are common in this population (65, 66, 68, 69). Cognitive symptoms may manifest subtly, with women reporting difficulties in decision-making, remembering medication schedules, or managing daily responsibilities. These impairments can undermine self-management of OP and increase reliance on caregivers, thereby reducing a woman’s sense of autonomy and self-efficacy (45). Emotional dysregulation, including mood instability, irritability, and low frustration tolerance, is another significant yet underreported outcome. These symptoms often reflect an overwhelmed psychological coping system, particularly in women who must simultaneously manage chronic illness, caregiving responsibilities, and social role transitions common in later life. Emotional dysregulation may also signal the presence of subclinical mood or anxiety disorders, which require targeted psychological support (70, 71).

### 4.3 Behavioral outcomes and impacts on daily life

The psychological burden of OP often leads to behavioral changes that can exacerbate disease progression and reduce quality of life (72). One of the most concerning behaviors is the reduction or cessation of physical activity due to fear of injury (73, 74). While cautiousness is understandable, complete avoidance of movement contributes to muscle atrophy, joint stiffness, and further bone demineralization. Over time, this leads to a downward spiral of physical and mental decline. Poor adherence to medication is another prevalent behavioral issue. Women experiencing depression, cognitive challenges, or emotional fatigue may forget doses, prematurely discontinue medications due to perceived ineffectiveness, or avoid treatment altogether out of fear of side effects (75). Suboptimal adherence compromises therapeutic outcomes and increases the risk of fractures and hospitalization (76). In addition, nutritional neglect—either through poor appetite or reduced motivation to prepare balanced meals—is common in women with depression or anxiety (77, 78). Deficiencies in calcium, vitamin D, and other nutrients further exacerbate bone loss and reduce overall vitality (79, 80). Substance use, such as smoking or excessive alcohol intake, may also be employed as maladaptive coping strategies, further contributing to both psychological and skeletal deterioration (81, 82). The cumulative result of these psychological and behavioral challenges is a marked reduction in HRQoL. Cross-sectional analytical researches have consistently shown that osteoporotic women report lower scores in both physical and mental health domains of standardized HRQoL assessments (83, 84). These include limitations in mobility, self-care, social functioning, and emotional well-being. Women who have suffered fractures often experience persistent pain, disfigurement, and a loss of independence, which can be profoundly demoralizing. Moreover, the loss of perceived social role—such as the ability to care for grandchildren, maintain a household, or participate in community life—can deeply affect identity and life satisfaction. These experiences underscore the necessity of addressing psychological and behavioral outcomes as core components of OP management, not merely secondary concerns.

## 5 Psychological interventions and management approaches

The emotional distress, cognitive burden, and behavioral changes that accompany OP necessitate a multidimensional approach that extends beyond pharmacotherapy. Effective psychological support not only alleviates mental health symptoms but also improves treatment adherence, physical functioning, and quality of life (85, 86). This section outlines a hierarchical, evidence-based management strategy starting with low-intensity measures and progressing to integrated specialist care, demonstrated effective in managing the complex interplay between psychological well-being and OP in postmenopausal women.

### 5.1 Health education and self-management support

Health education and self-management support represent the foundational, low-intensity tier of psychological care women living with OP (87–89). Education programs tailored to the specific needs and concerns of postmenopausal women can correct misinformation, reduce illness-related fear, and enhance self-efficacy in managing the disease. Effective programs typically cover key topics such as bone biology, the effects of menopause on skeletal health, fracture prevention strategies, the role of nutrition and physical activity, and medication adherence. Importantly, education should also address the emotional aspects of OP, helping women reframe the diagnosis as manageable rather than debilitating (90). When delivered through interactive formats—such as peer-led workshops, nurse-led counseling, or tele-education platforms—these interventions can facilitate meaningful engagement and behavioral change (91). Additionally, digital health tools and mobile applications, often incorporating AI-assisted monitoring, now offer scalable ways to reinforce learning, provide real-time reminders, and track health behaviors, enhancing autonomy and continuity of care (92, 93). Effectiveness is typically measured via validated questionnaires for self-efficacy, health literacy, and treatment adherence rates. Empowering women with knowledge and skills not only reduces psychological distress but also fosters active participation in health maintenance, ultimately leading to better long-term outcomes.

### 5.2 Cognitive behavioral therapy

Cognitive behavioral therapy (CBT) is a well-established, structured, and goal-oriented psychotherapeutic approach that has shown significant efficacy in treating depression, anxiety, and maladaptive behaviors in patients with chronic diseases, including OP (94, 95). In PMOP women, CBT targets distorted cognitions related to bodily fragility, perceived helplessness, and fear of falling or fracture (96). Through the process of cognitive restructuring, patients learn to identify and challenge negative automatic thoughts—such as catastrophic thinking about physical limitations—and replace them with more realistic, adaptive beliefs (97). Behavioral components of CBT encourage gradual exposure to avoided activities, promoting functional independence and engagement in physical activity, which is vital for bone health. High-level evidence, including systematic reviews and meta-analyses, confirms the robust efficacy of CBT in reducing the risk of relapse in major depressive disorder (98). Additionally, pre- to posttreatment case replication series have demonstrated that the benefits of CBT extend beyond sleep disorders to significantly reduce depressive symptoms and improve overall well-being (99). Although specific data on PMOP is limited, these findings suggest that CBT can reduce depressive symptoms, alleviate health-related anxiety, and improve overall psychological resilience in this population (98–100). Effectiveness in clinical trials is generally assessed using standardized psychometric scales, including the Beck Depression Inventory (BDI), generalized anxiety disorder-7, and validated HRQoL instruments (101–103). Furthermore, CBT may indirectly enhance physical outcomes by promoting better sleep, pain coping strategies, and medication adherence (104, 105). Its flexibility—being deliverable through in-person sessions, group formats, or digital platforms and telehealth—makes it particularly adaptable for older adults with mobility limitations or limited access to mental health professionals.

### 5.3 Mind-body interventions

Mind-body interventions, such as Tai Chi, Yoga, and mindfulness-based stress reduction (MBSR), are gaining increasing attention as non-pharmacological strategies that simultaneously address psychological and physical health dimensions in women with PMOP (106–109). A meta-analysis and trial sequential analysis of randomized controlled trial (RCT) indicates that Tai Chi, characterized by slow, deliberate movements combined with focused breathing and mental concentration, is a safe and optional exercise that may improve BMD loss in postmenopausal women, with greater benefits observed after six months of practice. Specifically, compared with non-intervention, Tai Chi was shown to significantly improve BMD of the lumbar spine (MD = 0.04; 95% CI 0.02 to 0.07) and femoral neck (MD = 0.04; 95% CI 0.02 to 0.06) (110). Yoga, similarly, offers a combination of physical postures, deep relaxation, and meditative awareness that can improve mood, promote flexibility, and reduce chronic musculoskeletal discomfort. A systematic review and meta-analysis evaluating Yoga and Pilates interventions on BMD found the pooled effect size to be non-significant compared to control (pooled effect size 0.07; 95% CI: -0.05 to 0.19) (111). However, the authors concluded that BMD maintenance in the postmenopausal population, combined with a beneficial impact on fracture risk factors such as strength and balance, constitutes a positive clinical outcome (111). Mindfulness-based interventions, including MBSR, help individuals develop nonjudgmental awareness of their bodily sensations and emotional states, which can be particularly helpful in reducing pain catastrophizing, improving psychological flexibility, and managing stress-related symptoms (109). Collectively, these practices enhance self-regulation, reduce physiological stress responses (e.g., cortisol levels), and foster a sense of agency over health. Effectiveness of these mind-body approaches is commonly evaluated through subjective reports of pain and anxiety (e.g., Visual Analog Scale/VAS, Hospital Anxiety and Depression Scale/HADS), improvements in balance (e.g., Berg Balance Scale/BBS), and enhanced HRQoL scores (112, 113). Importantly, when practiced in group settings, they also provide opportunities for social engagement and peer support, which are critical in mitigating loneliness and social isolation commonly experienced by older women. In contrast to mind-body practices like Tai Chi, which possess direct PMOP-specific RCT evidence regarding BMD maintenance and fall reduction, interventions such as Yoga and MBSR largely derive their support from broader chronic disease and menopausal cohorts, warranting further PMOP-dedicated clinical validation.

### 5.4 Multidisciplinary and holistic care models

Multidisciplinary care models represent the highest tier of integrated, specialist care for the management of OP in postmenopausal women, particularly when psychological health is prioritized alongside physical treatment (114, 115). Qualitative systematic reviews confirm that healthcare professionals advocate for these interprofessional collaboration models to optimize care and overcome treatment fragmentation (115). These models bring together a team of professionals—typically including endocrinologists or rheumatologists, clinical psychologists, physical therapists, dietitians, nurses, and social workers—who collaborate to develop individualized care plans addressing the biological, psychological, and social dimensions of health. In the context of multimorbidity, which frequently includes conditions like CVD and diabetes in this population, the multidisciplinary model is critical for coordinating complex care. This model facilitates careful oversight of polypharmacy (the use of multiple medications), which is a major concern as it can increase the risk of adverse drug interactions and falls (116). For example, a woman presenting with a recent fracture and depressive symptoms may receive concurrent fracture rehabilitation, CBT-based psychological support, dietary counseling to optimize calcium and vitamin D intake, and social services to address environmental safety and isolation. This integrated approach ensures that no aspect of her condition is overlooked and facilitates seamless communication between specialties, reducing care fragmentation. Furthermore, case managers or health coaches may assist in coordinating services, monitoring progress, and enhancing patient engagement. Evidence shows that such comprehensive care not only improves physical recovery and treatment adherence but also leads to significant reductions in depressive symptoms, fear of falling, and perceived disability (117). The effectiveness of these models is measured comprehensively via a range of clinical outcomes, including fracture recurrence rates, compliance with medication regimens, and standardized HRQoL and functional status assessments. In resource-limited settings, adapted versions of these models using community health workers or telemedicine can be effective alternatives, particularly when designed with cultural sensitivity and accessibility in mind.

### 5.5 Pharmacological support for psychological symptoms

While psychological interventions are often the first line of treatment for emotional and behavioral disturbances associated with OP, pharmacological therapies may be warranted for postmenopausal women with moderate to severe mood or anxiety disorders. Selective serotonin reuptake inhibitors (SSRIs) and serotonin-norepinephrine reuptake inhibitors (SNRIs) are commonly prescribed antidepressants that have demonstrated efficacy in this population. However, caution is required, as the association between long-term antidepressant use and skeletal integrity is complex. Evidence from a systematic review and meta-analysis (118) indicates a significant detrimental association between novel antidepressant use (especially SSRIs), decreased BMD, and a substantially increased risk of hip fracture (pooled OR 2.50; 95% CI 2.26–2.76). Furthermore, a large-scale cross-sectional analysis (119) found that antidepressant use was associated with a 44% increase in the odds of OP. Specifically, specialized cohort and self-controlled case series analyses (120) in menopausal women found SSRI/SNRI prescribing to be associated with a 32% increased risk of osteoporotic fractures (adjusted HR = 1.32; 95% CI 1.29–1.35), particularly with longer treatment duration. This association, though not conclusively causal, may be mediated by falls, altered neuromuscular function, or direct effects on bone metabolism. This risk is further compounded in patients with multimorbidity and polypharmacy, as the cumulative burden of multiple medications significantly elevates the likelihood of adverse drug-drug interactions and fall-related injuries (121). Therefore, the decision to initiate antidepressants must be individualized, considering the severity of psychiatric symptoms, comorbid conditions, and concurrent OP treatment. Collaboration between primary care physicians, psychiatrists, and OP specialists is essential to balance psychiatric benefits with skeletal safety. In all cases, pharmacotherapy should ideally be accompanied by psychological counseling or behavioral interventions to maximize therapeutic effectiveness and minimize reliance on medications alone. Critically, current pharmacological options primarily offer symptomatic relief; there is a notable lack of therapeutic agents designed to target shared underlying pathologies such as systemic oxidative stress or BDNF deficiency (122, 123).

Clinicians must carefully weigh antidepressant selection against skeletal safety. SSRIs generally exhibit a higher risk of accelerated BMD loss and fall-related fractures compared to SNRIs or atypical agents, with fracture risk increasing in a duration-dependent manner. Polypharmacy also heightens drug-drug interactions risks and orthostatic dizziness. Management recommendations include establishing baseline DXA prior to initiating prolonged SSRI therapy, co-prescribing bone-protective agents (e.g., bisphosphonates, calcium/vitamin D), and monitoring fall risk. Crucially, in cases of severe depression, the immediate psychiatric and life-saving benefits of antidepressant therapy clearly outweigh potential skeletal risks, provided proactive bone health management is implemented.

**Figure 4:**
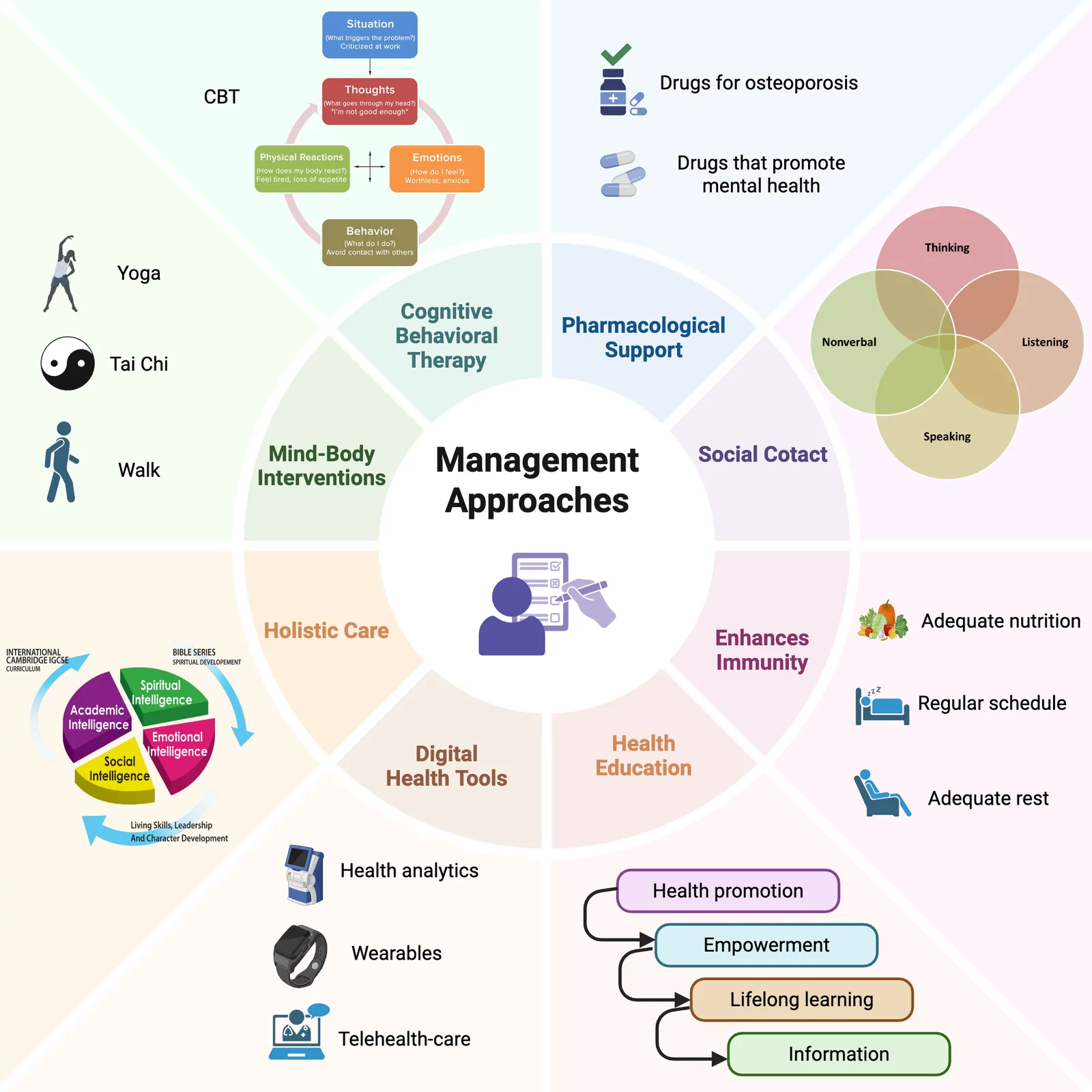
Comprehensive framework of psychological interventions and integrated management strategies for patients with postmenopausal osteoporosis (PMOP). This diagram provides a comprehensive overview of psychological interventions and management strategies for PMOP, addressing emotional distress, cognitive burden, and behavioral changes.

**Figure 4** provides a comprehensive overview of the psychological interventions and management approaches discussed in this section, highlighting their interconnectedness and multidimensional nature in addressing the complex interplay between psychological well-being and women with PMOP.

## 6 Global and sociocultural determinants of psychological health and care access

While our review highlights the bidirectional neurobiological mechanisms of PMOP, a comprehensive understanding requires integrating the profound influence of global and sociocultural determinants. Illness perception of OP varies dramatically across cultures; in many regions, it is often viewed as an inevitable, non-pathological consequence of aging, leading to fatalistic acceptance which hinders proactive psychiatric and orthopedic management. This perception is frequently compounded by the social stigma associated with physical frailty and declining function. Particularly in cultures where a woman’s self-worth is tightly tethered to active functional roles, stigma suppresses the open disclosure of psychological distress, often leading to the somatization of depressive and anxiety symptoms (124).

Furthermore, socioeconomic factors create significant variations in care access globally (125, 126). Women in low- and middle-income countries face systemic barriers, including limited DXA availability and the high cost of anti-osteoporotic medications, alongside a critical shortage of multidisciplinary mental health teams. This restricted access, combined with the financial burden of chronic disease, directly exacerbates allostatic load, escalating the risk of clinical anxiety and depression. In alignment with UN Sustainable Development Goals (SDG 3 and SDG 10), future efforts must prioritize cross-cultural, stratified research to develop culturally-adapted, economically feasible psychotherapeutic and somatic interventions to mitigate these global disparities in mental health equity.

## 7 Challenges, limitations, and future perspectives

### 7.1 Limitations of current evidence and review

While this review synthesizes a broad range of evidence, several limitations must be acknowledged. First, the current evidence base is dominated by cross-sectional designs, which complicates the establishment of definitive causality in the bone-mood axis. Second, there is significant heterogeneity in the psychometric tools used to assess distress, ranging from self-report scales to clinical interviews, which may affect the comparability of prevalence rates. Regarding the review itself, although a structured search was conducted, its narrative nature may entail a degree of selection bias compared to a formal PRISMA-compliant systematic review. Furthermore, the lack of standardized reporting for effect sizes across all interventions makes direct clinical comparisons challenging.

### 7.2 Existing challenges and research gaps

Existing literature reveals critical gaps in addressing the psychological burden of PMOP. First, there is a lack of large-scale, longitudinal studies mapping the precise temporal trajectory of this physical-mental interplay. While neurobiological links (e.g., BDNF, neuroinflammation, and oxidative stress) are increasingly recognized, a profound clinical gap remains: a lack of dual-action, biomarker-targeted therapies that can simultaneously ameliorate shared molecular pathways of skeletal and psychiatric degeneration, as current pharmacotherapy remains limited to isolated symptomatic relief. Second, specialized psychological therapies tailored for osteoporotic populations remain critically underdeveloped; few CBT protocols incorporate OP-specific cognitive distortions, such as the “fear of falling” or “kinesiophobia”, and rigorous evaluation of their cost-effectiveness is lacking. Third, existing studies persistently isolate psychological outcomes from broader multi-modal frameworks, highlighting an urgent need for integrated care models that evaluate the synergistic effects of pharmacotherapy, physical rehabilitation, and structured psychological support.

### 7.3 Future research agenda

Building upon these challenges, future research should prioritize identifying specific PNI or neuroendocrine biomarkers, such as BDNF levels and gut microbiome metabolites, that mediate the relationship between depression and bone turnover markers (e.g., CTX, P1NP) to reveal precise therapeutic targets. Simultaneously, it is essential to conduct randomized controlled trials to test the long-term clinical efficacy of innovative, integrated therapeutic approaches. Specifically, studies should assess whether embedding culturally-adapted CBT or mind-body interventions into routine somatic care yields a sustained reduction in both fracture risk and polypharmacy burden over at least 12 months. Addressing global disparities also requires quantifying how nurse-led, biopsychosocial screening protocols impact treatment adherence and psychiatric quality of life in low-resource settings. Ultimately, longitudinal cohorts spanning five or more years are imperative to determine the definitive impact of early psychosomatic screening on major osteoporotic fracture rates and all-cause mortality in newly diagnosed postmenopausal women.

## 8 Conclusion

Osteoporosis in postmenopausal women represents a quintessential psychosomatic challenge, capturing a profound interplay between a major physical disorder and a debilitating psychological burden. Evidence synthesized in this review underscores that psychological distress—particularly depressive and anxiety symptoms—is not merely a secondary reaction to functional decline, but a potent, independent risk factor that drives systemic pathophysiology, accelerates bone loss, and compromises clinical outcomes. These multifaceted impacts are orchestrated via complex biopsychosocial pathways, wherein neuroendocrine dysregulation and chronic systemic inflammation intersect with social stressors and maladaptive illness perceptions.

While evidence-based psychological therapies, such as cognitive-behavioral therapy and mindfulness-based interventions, show promising efficacy, a critical gap persists in clinical models targeting the shared molecular pathologies of the bone-mood axis. To bridge this divide, we advocate for a paradigm shift toward a comprehensive psychosomatic care model that integrates routine, early psychological screening into personalized skeletal management. Future research must leverage longitudinal designs and scalable digital mental health strategies to harmonize physical and psychological well-being, ultimately fostering holistic health equity and long-term quality of life for the aging female population worldwide.
